# Comparative effectiveness of floss band-augmented perceptual balance training on balance, proprioception, and lower limb function in women with chronic ankle instability: Implications for injury prevention and rehabilitation in public health

**DOI:** 10.3934/publichealth.2026034

**Published:** 2026-05-25

**Authors:** Melika Hamzehzadeh, Seyed Kazem Mosavi Sadati, Abdulrasol Daneshjoo, Hadi Mohammadi Nia Samakosh, Sameer Badri Al-Mhanna, Georgian Badicu

**Affiliations:** 1 Department of Sports Injuries and Corrective Exercises, Faculty of Humanities, Islamic Azad University, East Tehran Branch, Tehran 1866113118, Iran; 2 Department of Motor behavior, SR.C. Islamic Azad University, Tehran 1477893855, Iran; 3 Department of Biomechanics and Corrective Exercises and Sports Injuries, University of Kharazmi, Tehran 15719-14911, Iran; 4 Department of Physiology, Saveetha Medical College and Hospital, Saveetha Institute of Medical and Technical Sciences, Chennai, Tamil Nadu, India; 5 Department of Higher Studies, Al-Qasim Green University, Babylon, Iraq; 6 Department of Physical Education and Special Motricity, Faculty of Physical Education and Mountain Sports, Transilvania University of Braşov, Braşov, 500068, Romania

**Keywords:** chronic ankle instability, perceptual balance training, floss bands, balance, joint position sense, lower limb performance, women, injury prevention

## Abstract

In this study, an assessor-blinded randomized trial with concealed allocation was conducted. Thirty women aged 25–35 years with chronic ankle instability (CAIT score ≤ 24 on the affected ankle) were randomly assigned (1:1) to perceptual balance (PB) training with floss bands (FB group, n = 15) or PB training alone (n = 15). Both groups completed a 6-week program (3 sessions/week), progressing from static to dynamic and plyometric tasks. FBs were applied at perceived occlusion pressure of 5–7/10 (0–10 scale); no adverse events occurred. Outcomes (assessed on the affected limb only) included static balance (single-leg stance time), dynamic balance (Y-balance test–normalized reaches in anterior, posteromedial, and posterolateral directions and composite score), ankle proprioception [joint position sense (JPS) error at 20° plantarflexion], and functional performance (single-leg hop and triple-hop for distance). A 2 × 2 mixed ANOVA (time × group) was used, with Bonferroni post hoc tests. Effect sizes were partial η² (ANOVA interactions) and Cohen's d_z_ (repeated-measures within-group changes with 95% CI). Both groups improved significantly (p < 0.01). The FB group showed greater improvements in static balance (25.7 ± 2.9 s vs. 22.3 ± 2.8 s, p = 0.015), dynamic balance (composite and all directions, p < 0.015), JPS (4.2 ± 1.1° vs. 6.1 ± 1.3°, p = 0.022), single-leg hop (1.80 ± 0.15 m vs. 1.65 ± 0.18 m, p = 0.032), and triple-hop (5.10 ± 0.30 m vs. 4.70 ± 0.35 m, p = 0.039). Interaction effect sizes (dz) ranged from 0.143 to 0.234 (large). These findings suggest that floss band augmentation may provide additional short-term benefit to PB training in women with CAI, although mechanisms remain speculative, and larger, longitudinal trials are needed.

## 1. Introduction

Chronic ankle instability (CAI) is a prevalent musculoskeletal condition characterized by recurrent episodes of ankle “giving way”, persistent pain, reduced joint position sense (JPS), and impaired balance, often following an initial ankle sprain [1]. It affects approximately 30%–70% of individuals who experience lateral ankle sprains, with a higher incidence among women due to biomechanical factors such as increased Q-angle, greater joint laxity, and hormonal influences like estrogen on ligament integrity [2]. Recent evidence also highlights sex-specific differences in neuromuscular control and movement patterns that may increase lower limb injury vulnerability in females, supporting the need for targeted protocols in women with CAI [3]. This gender disparity exacerbates the public health burden, as CAI contributes to decreased physical activity, heightened fall risk, and long-term functional limitations, potentially leading to secondary conditions like osteoarthritis [4]. In women aged 25–35 years, who are often active in sports or daily activities, CAI not only hampers performance but also impacts quality of life, emphasizing the need for targeted rehabilitation strategies [5],[6].

Rehabilitation for CAI typically focuses on restoring sensorimotor function, including balance, JPS, and lower limb performance, to prevent recurrence and enhance stability [7],[8]. Perceptual balance (PB) training, which integrates cognitive and sensory challenges to stimulate visual, vestibular, and proprioceptive systems, has emerged as a promising approach [9],[10]. Recent umbrella reviews confirm moderate effects of balance training on dynamic postural control in CAI (Hedges' g ≈ 0.91), though with high heterogeneity and low-to-moderate evidence certainty [11]. This training enhances neuromuscular coordination by incorporating tasks like single-leg stands on unstable surfaces or eyes-closed exercises, thereby improving postural control and reducing instability symptoms [12],[13]. Studies have demonstrated its efficacy in CAI populations; for instance, Kim et al. (2022) conducted a randomized trial showing that neuromuscular and strength-based protocols significantly improved pathomechanical, sensory-perceptual, and motor-behavioral impairments in CAI patients [14]. Similarly, Wang et al. (2023) conducted a meta-analysis and found that dual-task balance training reduced instability and injury risk [15]. However, these interventions often lack adjunctive tools to amplify effects, particularly in women, where hormonal and anatomical differences may influence outcomes [16].

Recent innovations in rehabilitation include the use of floss bands (FB), elastic compression devices that apply intermittent pressure to soft tissues, promoting blood flow restriction (BFR) and subsequent reperfusion to enhance tissue mobility, proprioceptive feedback, and muscle activation [17],[18]. FBs work by compressing tissues temporarily, stimulating mechanoreceptors, and improving joint range of motion (ROM), which is crucial for ankle stability [19],[20]. Early research on FBs has shown benefits in various contexts. Driller and Overmayer (2017) reported immediate improvements in ankle dorsiflexion ROM and jump performance post-flossing [19]. In a chronic stroke population, Moon and Kim (2024) found that floss bands enhanced ankle ROM, balance, and gait, suggesting applicability to instability conditions [21]. Cheatham et al. (2024) conducted a systematic review and highlighted FBs' role in athletic performance, noting moderate increases in ankle ROM and potential for balance gains, though evidence on strength remains inconsistent [22].

Despite these advances, the integration of floss bands with PB training for CAI remains underexplored. Existing studies on FBs often focus on acute effects or isolated applications, such as in healthy athletes or post-sprain recovery. For example, Rosier (2022) examined long-term effects of tissue flossing on ankle dorsiflexion in CAI athletes, finding sustained ROM improvements but limited data on balance or JPS [23]. Gao et al. (2024) proposed mechanisms like reactive hyperemia and neural modulation for FB's efficacy in sports rehabilitation [24]. In CAI-specific research, Moon and Kim (2022) demonstrated FBs' positive impact on ankle ROM and balance ability, but without perceptual elements [21]. Combined interventions, such as strength and balance training, have shown promise; Yilmaz et al. (2024), in a systematic review, confirmed proprioceptive training's benefits on sports performance, yet floss augmentation was absent [12].

Notably, few studies address gender-specific responses, despite women's heightened vulnerability to CAI [25]. Alahmari et al. (2021) reported that combined strengthening and proprioceptive training improved stability in CAI across ages, but did not stratify by sex [26]. Fakontis et al. (2023), in a meta-analysis, found that resistance training with elastic bands enhanced balance in CAI, suggesting potential for FBs as a similar tool, though direct comparisons are scarce [27]. A pilot study by Chang et al. (2021) indicated that FBs improved thigh flexibility and balance without hindering JPS, but focused on knees rather than ankles [28]. In women, Hahn (2009) highlighted sex differences in ankle stability, underscoring the need for tailored protocols [29].

This study innovates by comparing PB training with and without FBs in women with CAI, addressing gaps in combined modalities and gender focus. Previous research lacks rigorous comparisons of floss-augmented perceptual training on multifaceted outcomes like static/dynamic balance, JPS at specific angles (e.g., 20° plantarflexion), and lower limb function via hop tests [30],[31]. By incorporating FBs' BFR-like effects into perceptual exercises, this intervention may enhance sensory integration and neuromuscular efficiency more than training alone, potentially offering a cost-effective, accessible tool for rehabilitation [32],[33]. The novelty lies in its quasi-experimental design, targeting women aged 25–35, and evaluating progressive protocols from stability to plyometrics, which could inform public health strategies for injury prevention [34].

Recent systematic evidence supports the effectiveness of balance training for improving postural control in CAI, although heterogeneity is high and evidence certainty is low to moderate [11]. Proximal (hip/knee) strength deficits are also associated with CAI, suggesting a role for kinetic chain assessment in comprehensive rehabilitation [35]. Sex-specific neuromuscular and landing differences may contribute to higher lower-limb injury vulnerability in women [3]. The primary aim was to compare the effects of 6-week PB training with those without FB augmentation on balance, JPS, and hop performance in women with CAI. We hypothesized that the FB-augmented group would show significantly greater improvements across all outcomes.

## 2. Materials and methods

### 2.1. Study design

**Figure 1.**
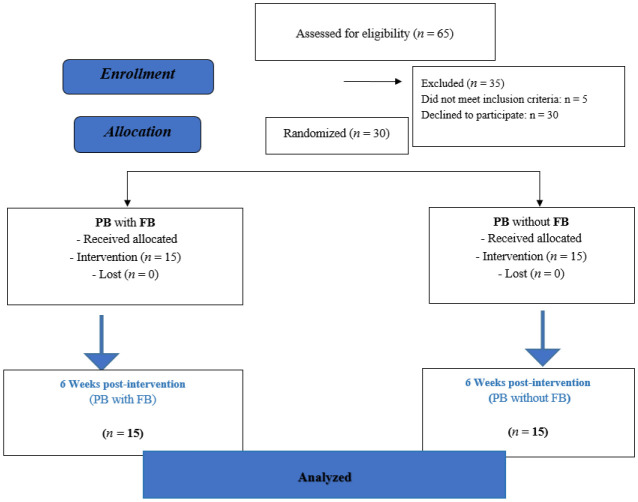
Flow diagram.

This study employed a randomized allocation design with assessor blinding and concealed allocation using sealed opaque envelopes, in line with key elements of CONSORT guidelines for randomized trials. Due to severe administrative and operational disruptions at the Iranian Registry of Clinical Trials (IRCT) during the study initiation period (July–September 2025), which coincided with escalating regional geopolitical tensions, military escalation, and conflict-related impacts on governmental services in Iran (including reported disruptions to public administrative systems amid the ongoing regional conflict starting February 2026), our submitted prospective registration application was neither processed nor assigned a trial code despite repeated follow-up attempts. This external constraint prevented formal prospective registration. Ethical approval was obtained from the Institutional Review Board of the Islamic Azad University, East Tehran Branch (Ethics Code: SSRI.REC-2504–2946), and all procedures adhered to the Declaration of Helsinki. A CONSORT-style flow diagram is provided as Figure 1. Analysis was per-protocol (no dropouts).

### 2.2. Participants

A total of 30 women aged 25–35 years with CAI were screened for eligibility through purposive sampling at the sports club. Participants were divided into two groups: PB training with FB (n = 15) and PB training without FB (non-FB) (n = 15). Participants were included based on the following criteria: (1) a Cumberland Ankle Instability Tool (CAIT) score ≤ 24 on the affected ankle (validated cutoff) [36] (baseline CAIT: 18.60 ± 4.22 for the FB group and 19.15 ± 4.60 for the PB-only group); (2) a history of at least 2–3 episodes of perceived ankle instability (“giving way”) in the past 12 months; (3) no lower limb surgery or acute ankle injuries within the past 6 months [8]; (4) absence of neurological, vestibular, or other musculoskeletal disorders impacting balance; and (5) commitment to a 6-week training program. Exclusion criteria included: (1) pregnancy, (2) cardiovascular or peripheral vascular diseases contraindicating FB application, (3) concurrent participation in other rehabilitation or exercise programs, and (4) severe pain or instability preventing physical testing [26],[37].

### 2.3. Sample size

A priori power analysis (GPower 3.1) for 2 × 2 mixed ANOVA (f = 0.25, α = 0.05, power = 0.80) indicated 13–15 participants per group (allowing ~10% attrition).

### 2.4. Outcomes

All assessments were conducted pre- and post-intervention by one certified physiotherapist blinded to group allocation to minimize bias. Tests were performed in a controlled indoor gym environment (temperature 22–24 °C, stable lighting, non-slip flooring) to ensure consistency. Participants wore standardized athletic shoes to reduce variability. A familiarization session was held one week prior to baseline testing to familiarize participants with the procedures, minimizing learning effects. Assessments were conducted in the following order to prevent fatigue: JPS, static balance, dynamic balance, and lower limb performance, with 5-min rest intervals between tests. All primary outcomes were performed on the affected limb only. Y-balance distances were normalized to limb length, with the composite score reported as (sum of three normalized reaches/3) × 100. The measured outcome measures are described below.

#### 2.4.1. Static balance (single-leg balance test)

Participants were instructed to stand on the affected leg, lifting the non-weight-bearing leg so that the knee was flexed and the foot did not contact the ground. Hands were placed on the hips, and participants closed their eyes to eliminate visual feedback. They were tasked with maintaining balance without falling or touching the ground with the non-weight-bearing foot. A stopwatch was used to record the duration (seconds) that participants maintained the position, with the test ending upon loss of balance (e.g., ground contact, hand movement, or excessive swaying). The test was repeated on the contralateral leg, and the average of three trials per leg was recorded. Normative data indicate that for individuals aged 20–39 years, optimal performance is >24 seconds [26]. The single-leg balance test with eyes closed is a validated measure of static balance in CAI populations, showing high test-retest reliability (ICC = 0.87–0.91) and concurrent validity with force plate measures (r = 0.82) for detecting postural control deficits [38].

#### 2.4.2. Dynamic balance (Y-balance test)

The Y-balance test assessed dynamic balance by measuring reach distances in anterior, posteromedial, and posterolateral directions. Leg length was measured twice per leg (from the anterior superior iliac spine to the medial malleolus) with participants lying supine, and the average was used to normalize reach distances (% leg length). Participants stood at the center of a Y-shaped grid on the affected leg, reaching with the free leg in each direction while maintaining balance. The big toe touched the furthest point possible along a tape measure, and the distance was recorded (cm). Three trials per direction were completed sequentially (clockwise or counterclockwise) after 2–3 practice trials. Participants returned to a bipedal stance between trials, resting for 10–15 s, with 30 s between trials and 60 s when switching directions. Trials were invalid if the balance was lost or the foot touched the ground before returning to the starting position [8]. The Y-balance test is a reliable and valid tool for assessing dynamic balance in CAI, with excellent test-retest reliability (ICC = 0.85–0.93 across directions) and high sensitivity (0.89) for detecting functional deficits [8].

#### 2.4.3. Joint position sense (Angle Meter 360 App)

JPS was measured using a validated smartphone inclinometer application (Angle Meter 360 mobile application, Android, v2.3.1); the device was secured on the foot dorsum with its axis aligned to the lateral malleolus. Verbal and visual cues minimized hip/knee compensation. Participants sat with their eyes closed to eliminate visual feedback. The examiner passively moved the ankle to 20° plantarflexion, held it for 5 s to allow perception, and returned it to neutral. Participants then actively reproduced the 20° angle. The smartphone, secured to the lateral ankle with a strap, recorded the reproduced angle. Absolute error (degrees) between the target and reproduced angles was calculated, with three trials averaged. Errors <5° are considered normal, while errors >10° may indicate proprioceptive deficits due to CAI [8]. The Angle Meter 360 app is a valid and reliable tool for ankle JPS, with good reliability (ICC = 0.88) and concurrent validity against goniometry (r = 0.85) in clinical populations [39],[40].

#### 2.4.4. Lower limb performance (single-leg and triple-hop tests)

The single-leg hop test involved participants placing their hands on their hips, positioning the big toe of the affected leg behind a starting line on a 2-m tape measure, and hopping forward as far as possible while landing stably. The distance (meters) from the starting line to the heel was recorded. The triple-hop test required three consecutive hops on the affected leg without loss of balance, with the total distance measured. Three trials per test were averaged. The limb symmetry index (LSI) was calculated as (affected leg distance / unaffected leg distance) × 100, with LSI < 90% indicating asymmetry [8],[27]. Single-leg and triple-hop tests are valid for assessing lower limb function in CAI, with high reliability (single-hop ICC = 0.96; triple-hop ICC = 0.95) and criterion validity against functional performance tasks (r = 0.79–0.84) [14].

#### 2.4.5. Floss band protocol

**Figure 2.**
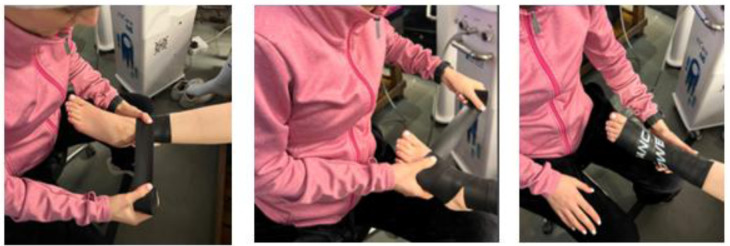
Apply a bandage to the ankle.

Both groups completed a 6-week PB training program (3 sessions/week, 45 minutes/session), supervised by certified physiotherapists. The protocol progressed from stability-focused exercises (weeks 1–2) to dynamic tasks (weeks 3–4) and plyometric movements (weeks 5–6), targeting sensorimotor integration (visual, vestibular, proprioceptive) [10]. Each session included a 5-min warm-up (light jogging, ankle circles) and a 5-min cool-down (stretching). Exercises were performed on the affected leg, with the unaffected leg used only during warm-up. To account for menstrual cycle influences on performance and injury risk in female participants, sessions were scheduled and adjusted based on self-reported menstrual phase tracking using a standardized app (e.g., Clue or Flo). During the menstrual phase (days 1–5 of the cycle), exercise intensity was reduced by 20%–30% if participants reported symptoms such as cramps, fatigue, or increased joint laxity, which are common due to fluctuating estrogen and progesterone levels [4],[14]. Specific accommodations included shortening exercise durations (e.g., from 1–2 min to 45–60 s per set), incorporating additional rest periods (up to 2 min between exercises), and prioritizing low-impact perceptual tasks over plyometrics to minimize strain on the ankle. Participants were instructed to avoid sessions if severe pain (VAS > 5/10) or heavy bleeding occurred, rescheduling within the week to maintain compliance. Hydration, nutrition (e.g., iron-rich foods), and pain management strategies (e.g., heat application or over-the-counter analgesics, if approved by a physician) were recommended to support recovery. Menstrual symptoms were monitored via a weekly diary to ensure safety and optimize training efficacy, aligning with gender-specific guidelines for reducing CAI exacerbation during hormonal fluctuations [41]. The FB group used elastic FBs (Chinese-manufactured, 5 cm wide, 2 mm thick, black color) (Figure 2) applied to the ankle at 50%–70% perceived occlusion pressure for 1–2 min per exercise, followed by a 1-min release to enhance reperfusion and sensory feedback [20]. Elastic FBs were applied as follows: circumferential application from proximal lateral malleolus to distal calf (8–10 overlapping turns at moderate tension); participant-rated perceived pressure 5–7/10 on 0–10 scale; continuous monitoring by trained therapist; immediate removal if numbness, pain, or cyanosis occurred. The non-FB group performed identical exercises without FBs. Compliance was monitored, requiring ≥80% attendance. No adverse events or dropouts occurred. The protocol is detailed in Table 1.

Menstrual cycle was tracked descriptively via self-report mobile applications for descriptive purposes only; no formal statistical adjustment or subgroup analysis was performed due to sample size constraints.

**Table 1.** Perceptual balance training protocol.

Week	Session focus	Exercises	Sets/reps/duration	FB group
1–2	Stability	- Single-leg stand (eyes open/closed) - Balance board (static) - Toe-touch reach	3 sets × 30–60 s each 3 sets × 10 reps 3 sets × 12 reps	1–2 min per exercise, 1 min release
3–4	Dynamic	- Single-leg stand on foam pad - Lateral step-overs - Dual-task (counting backward while balancing)	3 sets × 45 s 3 sets × 12 reps 3 sets × 30 s	1–2 min per exercise, 1 min release
5–6	Plyometric	- Single-leg hops (forward/lateral) - Balance board (dynamic) - Controlled drop jumps	3 sets × 10 reps 3 sets × 30 s 3 sets × 8 reps	1–2 min per exercise, 1 min release

### 2.5. Randomization and allocation

Participants were randomly assigned to either the intervention group (PB training with FB) or the control group (PB training without FB) using a random number generator (RNG) implemented in Microsoft Excel software. A simple randomization method was employed, whereby each participant received a unique identifier, and the RNG generated a sequence that determined group allocation. To ensure balance between groups, the randomization sequence was reviewed to confirm an approximately equal distribution (15 participants per group). An independent researcher, blinded to the participants' baseline characteristics, performed the randomization to minimize allocation bias. Group assignments were concealed in sealed, opaque envelopes and revealed to participants only after the completion of baseline assessments. The physiotherapist responsible for delivering the intervention (PB training with or without FB) was aware of the group assignments, but the outcome assessors (responsible for measuring balance, JPS, and lower limb performance) were blinded. Collected data were coded using unique identifiers to conceal group allocation. To assess the effectiveness of blinding, participants completed a questionnaire post-intervention asking whether they believed they were in the intervention or control group, and the responses were analyzed to confirm successful blinding.

### 2.6. Statistical analysis

Data were analyzed using SPSS version 27 (IBM Corp., Armonk, NY, USA). Normality (Shapiro–Wilk) and homogeneity of variance (Levene's test) were verified for all variables. Descriptive statistics (mean ± standard deviation) were calculated for all variables at pre- and post-intervention. To examine changes over time and between groups, a 2 × 2 mixed-model ANOVA was conducted for each variable, with time (pre- vs. post-intervention) as the within-subjects factor and group (FB vs. non-FB) as the between-subjects factor. This model tested the main effects of time (improvements across both groups), group (differences between FB and non-FB groups), and the group × time interaction (differential improvements between groups). Greenhouse–Geisser corrections were applied if sphericity assumptions were violated (Mauchly's test, p < 0.05). Within-group effect sizes were calculated as Cohen's d_z_ (mean change/SD of change scores) with 95% CI. Effect sizes were reported as Cohen's d_z_ (d) for ANOVA effects (small: 0.01, medium: 0.06, large: 0.14) [42] and Cohen's d_z_ for within-group changes (small: 0.20, medium: 0.50, large: 0.80) [43], calculated as (post-mean - pre-mean) / pooled standard deviation. Statistical significance was set at p ≤ 0.05, and all analyses were two-tailed. No minimal clinically important difference (MCID) values are established for all outcomes in this population.

## 3. Results

### 3.1. Baseline characteristics

The descriptive statistics for the demographic and clinical characteristics of the participants are presented in Table 2. This table provides the means and standard deviations (SD) for age, height, weight, body mass index (BMI), time since last injury, CAIT score, and lower limb length for both groups. The results of an independent t-test indicated no significant differences between the two groups across these variables (p > 0.05), confirming baseline homogeneity and comparable distribution of characteristics prior to the intervention.

**Table 2.** Descriptive statistics of demographic and clinical variables.

Variable	Group	Mean ± SD	T	P-value
Age (years)	PB with FB	28.40 ± 2.77	0.44	0.66
	PB without FB	27.93 ± 3.01		
Height (m)	PB with FB	1.70 ± 0.09	−0.79	0.43
	PB without FB	1.72 ± 0.06		
Weight (kg)	PB with FB	61.46 ± 13.62	0.22	0.82
	PB without FB	60.53 ± 9.04		
BMI (kg/m²)	PB with FB	20.97 ± 5.58	−0.03	0.96
	PB without FB	21.04 ± 3.79		
Time since last injury (months)	PB with FB	9.00 ± 1.25	0.26	0.79
	PB without FB	8.86 ± 1.50		
CAIT score	PB with FB	25.00 ± 1.30	−1.92	0.06
	PB without FB	25.86 ± 1.06		
Lower limb length (cm)	PB with FB	94.45 ± 3.26	0.47	0.63
	PB without FB	94.97 ± 2.67		

**Table 3.** Pre-post + ANOVA outcome measures with effect sizes and 95% CIs.

Variable	FB group			PB without FB			p-value (group × time)
	Pre (mean ± SD)	Post (mean ± SD)	p-value (d, 95% CI)	Pre (mean ± SD)	Post (mean ± SD)	p-value (d, 95% CI)	
Static balance (s)	18.40 ± 3.20	25.70 ± 2.90	<0.001* (2.34, [1.82, 2.86])	18.10 ± 3.00	22.30 ± 2.80	0.002 (1.38, [0.92, 1.84])	0.015*
Y-anterior (%)	52.30 ± 4.12	64.80 ± 3.70	<0.001* (3.12, [2.52, 3.72])	51.90 ± 4.00	58.40 ± 3.87	0.003 (1.65, [1.15, 2.15])	0.012*
Y-posteromedial (%)	54.60 ± 4.50	67.90 ± 3.90	<0.001* (2.98, [2.40, 3.56])	54.20 ± 4.70	60.70 ± 4.10	0.004 (1.52, [1.04, 2.00])	0.007*
Y-posterolateral (%)	53.20 ± 4.32	66.10 ± 4.00	<0.001* (3.05, [2.46, 3.64])	52.80 ± 4.50	59.30 ± 4.01	0.005 (1.48, [1.01, 1.95])	0.014*
Y-composite (%)	53.36 ± 4.10	66.26 ± 3.68	<0.001* (2.45, [2.20, 3.20])	52.96 ± 4.10	59.46 ± 4.03	0.005 (1.46, [1.06, 1.99])	0.013*
JPS (°)	8.70 ± 1.60	4.20 ± 1.11	<0.001* (2.89, [2.32, 3.46])	8.9 ± 1.71	6.1 ± 1.31	0.006 (1.71, [1.20, 2.22])	0.022*
Single-leg hop (m)	1.50 ± 0.20	1.80 ± 0.15	<0.001* (1.67, [1.18, 2.16])	1.48 ± 0.22	1.65 ± 0.18	0.007 (0.87, [0.42, 1.32])	0.012*
Triple-hop (m)	4.20 ± 0.35	5.10 ± 0.30	<0.001* (2.43, [1.88, 2.98])	4.15 ± 0.40	4.70 ± 0.35	0.008 (1.23, [0.74, 1.72])	0.020*

Note: s, seconds; %, percentage; °, degree; m, meter; CI, confidence interval; D, effect size; *significant differences between pre- and post-tests.

### 3.2. Main effects and interaction

The 2 × 2 mixed-model ANOVA showed significant main effects of time (p < 0.001 for all outcomes), indicating improvements in both groups from pre- to post-intervention. A significant main effect of group was found for dynamic balance [anterior: F(1,28) = 6.32, p = 0.018, d = 0.184; posteromedial: F(1,28) = 7.89, p = 0.009, d = 0.220; posterolateral: F(1,28) = 5.47, p = 0.027, d = 0.163, and Y-composite: F(1,28) = 5.68, p = 0.024, d = 0.163], JPS [F(1,28) = 4.91, p = 0.035, d = 0.149], single-leg hop [F(1,28) = 5.12, p = 0.032, d = 0.155], and triple-hop [F(1,28) = 4.68, p = 0.039, d = 0.143], favoring the FB group. Significant group × time interactions were observed for static balance [F(1,28) = 6.75, p = 0.015, d = 0.194], dynamic balance [anterior: F(1,28) = 7.23, p = 0.012, d = 0.205; posteromedial: F(1,28) = 8.56, p = 0.007, d = 0.234; posterolateral: F(1,28) = 6.91, p = 0.014, d = 0.198; Y-composite: F(1,28) = 6.99, p = 0.01, d = 0.21], JPS [F(1,28) = 5.89, p = 0.022, d = 0.174], single-leg hop [F(1,28) = 7.14, p = 0.012, d = 0.203], and triple-hop [F(1,28) = 6.03, p = 0.020, d = 0.177], indicating greater improvements in the FB group over time.

### 3.3. Within-group changes

As shown in Table 3, the FB group significantly improved in static balance (p < 0.001, d = 2.34, 95% CI [1.82, 2.86]), dynamic balance (anterior: p < 0.001, d = 3.12, 95% CI [2.52, 3.72]; posteromedial: p < 0.001, d = 2.98, 95% CI [2.40, 3.56]; posterolateral: p < 0.001, d = 3.05, 95% CI [2.46, 3.64]; Y-composite: p < 0.001, d = 2.45, 95% CI [2.20, 3.20]), JPS (p < 0.001, d = 2.89, 95% CI [2.32, 3.46]), single-leg hop (p < 0.001, d = 1.67, 95% CI [1.18, 2.16]), and triple-hop (p < 0.001, d = 2.43, 95% CI [1.88, 2.98]). The non-FB group also improved significantly: static balance (p = 0.002, d = 1.38, 95% CI [0.92, 1.84]), dynamic balance (anterior: p = 0.003, d = 1.65, 95% CI [1.15, 2.15]; posteromedial: d = 1.52, 95% CI [1.04, 2.00]; posterolateral: p = 0.005, d = 1.48, 95% CI [1.01, 1.95]; Y-composite: p = 0.005, d = 1.46, 95% CI [1.06, 1.99]), JPS (p = 0.006, d = 1.71, 95% CI [1.20, 2.22]), single-leg hop (p = 0.007, d = 0.87, 95% CI [0.42, 1.32]), and triple-hop (p = 0.008, d = 1.23, 95% CI [0.74, 1.72]). Large within-group improvements occurred in both arms (dz > 1.2–3.1), consistent with the established efficacy of PB training. Significant group × time interactions (dz; 0.143–0.234) indicate that FB provided additional benefit, although the small sample size raises the risk of effect overestimation.

### 3.4. Between-group differences

Post hoc analyses with Bonferroni adjustments revealed that the FB group outperformed the non-FB group post-intervention in static balance (p = 0.015), dynamic balance (anterior: p = 0.012; posteromedial: p = 0.007; posterolateral: p = 0.014; Y-composite: p = 0.013), JPS (p = 0.022), single-leg hop (1.80 ± 0.15 m vs. 1.65 ± 0.18 m, p = 0.032), and triple-hop (5.10 ± 0.30 m vs. 4.70 ± 0.35 m, p = 0.039), consistent with significant interaction effects and highlighting the additional benefit of FBs.

## 4. Discussion

The present study investigated the comparative effectiveness of PB training with and without FB on balance, JPS, and lower limb performance in women aged 25–35 years with CAI. Both groups demonstrated significant improvements across all outcomes following the 6-week intervention, but the FB-augmented group showed superior gains, as evidenced by significant group × time interactions (p < 0.05) and large effect sizes (d = 0.143–0.234). These findings support the hypothesis that FB integration enhances PB training's benefits, likely through mechanisms such as intermittent compression, reperfusion, and mechanoreceptor stimulation, which amplify sensory integration and neuromuscular efficiency in a gender-specific CAI cohort [1],[2]. The following sections discuss the effects on each outcome separately, incorporating evidence from the study and recent literature.

### 4.1. Effects on balance

Balance improvements were a key outcome, with the FB group outperforming the non-FB group in both static and dynamic measures. For static balance (single-leg balance test), the FB group improved compared to the non-FB group, with a significant interaction [F(1,28) = 6.75, p = 0.015, d = 0.194]. This suggests FB's role in enhancing postural control via improved proprioceptive feedback and tissue mobility [19]. Consistent with this, a 2024 systematic review by Cheatham et al. (2024) reported moderate balance gains from tissue flossing in athletic populations, attributing effects to increased mechanoreceptor activation and reperfusion [22]. Similarly, Moon and Kim (2024) found floss bands improved balance in chronic stroke patients, with mechanisms like reactive hyperemia supporting our CAI-specific results [44]. A recent RCT by Bermúdez-Egidos et al. (2025) on flossing protocols in recurrent ankle sprains echoed these findings, showing enhanced static balance through manual therapy integration, though effects were more pronounced in our progressive PB protocol [45]. However, Wu et al. (2022) reported no acute balance changes with FB in healthy females, possibly due to the lack of a training component, contrasting our chronic intervention [46].

Dynamic balance (Y-balance test) also favored the FB group, with greater reaches in anterior (64.8% ± 3.7% vs. 58.4% ± 3.8%), posteromedial (67.9% ± 3.9% vs. 60.7% ± 4.1%), and posterolateral (66.1% ± 4.0% vs. 59.3% ± 4.0%) directions (p < 0.015, d > 0.198). This aligns with the meta-analysis by Wang et al. (2023) on dual-task training reducing CAI instability, where adjunctive tools like FB enhanced neuromuscular coordination [15]. Recent evidence from a systematic review by Tedeschi and Giorgi (2024) confirmed tissue flossing's positive impact on dynamic performance, including balance, via improved joint ROM and muscle function [47]. Gao et al. (2024) proposed neural modulation as a mechanism, supporting our results in women with CAI, who may benefit more due to biomechanical vulnerabilities [24]. Inconsistent findings from Chang et al. (2021) showed FB benefits for knee but not ankle dynamics, highlighting joint-specific effects; our ankle-focused PB-FB synergy likely explains the superior gains [28]. Overall, FB augmentation appears to amplify PB training's effects on balance, particularly in dynamic contexts relevant to injury prevention in CAI [8].

### 4.2. Effects on JPS

JPS, assessed as absolute error at 20° plantarflexion, improved more in the FB group (from 8.7 ± 1.6° to 4.2 ± 1.1°, d = 2.89) than the non-FB group (8.9 ± 1.7° to 6.1 ± 1.3°, d = 1.71), with a significant interaction (p = 0.022, d = 0.174). This indicates that FB's compression enhances JPS through mechanoreceptor stimulation and reperfusion [33]. Yilmaz et al. (2024)'s systematic review on proprioceptive training supports this, noting benefits on sports performance that could be augmented by tools like FB [12]. A 2025 study by Maemichi et al. elucidated flossing effects on joint ROM via tissue gliding, indirectly improving JPS in instability conditions [48]. Peng et al. (2024) linked JPS deficits to CAI severity, and our findings suggest FB mitigates this, especially in women with hormonal influences on ligament integrity [30]. Recent evidence from a 2025 study on proprioceptive neuromuscular facilitation in CAI showed similar improvements in JPS and balance, but our FB-PB combination yielded larger effects, possibly due to BFR-like mechanisms [49]. Contrasting results from Cheatham and Baker (2024) indicated limited long-term JPS gains with FB alone, emphasizing the need for integrated training as in our protocol [20]. FB augmentation likely enhances mechanoreceptor stimulation and sensory integration, as supported by broader literature on altered sensory processing [11]. These short-term gains may contribute to reduced long-term burden in chronic musculoskeletal conditions, as evidenced by large cohort studies linking environmental factors to persistent pain and referrals [50]. Hahn (2009) highlighted sex differences in ankle stability, suggesting women's greater laxity may amplify FB's sensory benefits [29]. Thus, FB-augmented PB training offers a promising approach to restoring proprioceptive deficits in female CAI patients.

### 4.3. Effects on lower limb performance

Lower limb performance in the FB group, evaluated via single-leg (1.50 ± 0.20 m to 1.80 ± 0.15 m, d = 1.67) and triple-hop tests (4.20 ± 0.35 m to 5.10 ± 0.30 m, d = 2.43), surpassed that of the non-FB group (p < 0.039, d > 0.143). This reflects FB's enhancement of muscle activation and ROM, synergizing with PB's plyometric progression. Kim et al. (2022) conducted a randomized controlled trial and demonstrated neuromuscular training's benefits on motor impairments in CAI, with FB potentially adding reperfusion effects [14]. Driller and Overmayer (2017) reported immediate jump gains post-FB, consistent with our chronic improvements [19]. A follow-up study on tissue flossing confirmed sustained effects on ankle ROM, jump, and sprint performance, supporting our hop test results in CAI [51]. Cheatham et al. (2024) noted tissue flossing's moderate increases in performance measures, though inconsistent for strength; our integrated approach may resolve this [20]. Lower limb performance improvements via hop tests align with proximal deficits (hip/knee strength) commonly associated with CAI, emphasizing a regional interdependence model [35]. Incongruent findings from Pisz et al. (2020) showed variable power jump effects, likely due to acute application vs. our 6-week protocol [52]. Fort-Vanmeerhaeghe et al. (2019) noted sex differences in landing deficits, implying FB's utility in female CAI for injury prevention [6]. Overall, FB enhances PB training's impact on functional performance, crucial for rehabilitation. Mechanistically, FB's BFR-like effects may reduce tissue adhesions and boost neuromuscular efficiency, particularly beneficial for women with CAI [27],[32]. This addresses gaps in prior research, which often neglected gender or combined interventions [26].

### 4.4. Study limitations

While the current quasi-experimental design provided valuable insights into the comparative effects of PB training with and without FB in women with CAI, several limitations must be acknowledged that may influence the interpretation and generalizability of the findings. First, the quasi-experimental nature of the study, without a true no-intervention control group, limits causal inferences. Although both groups received active PB training, the absence of a passive control group (e.g., no training or standard care) prevents isolation of the specific contributions of PB training alone or FB augmentation from natural recovery or placebo effects common in CAI rehabilitation. This design choice was driven by ethical considerations, as withholding rehabilitation from symptomatic participants could exacerbate instability risks, but it aligns with common challenges in rehabilitation trials where withholding care is impractical. Future randomized controlled trials (RCTs) with a sham or waitlist control could address this gap, as highlighted in systematic reviews emphasizing the need for robust controls in CAI studies [53],[54]. Second, the sample size (n = 30, with 15 per group) was relatively small, a frequent constraint in CAI rehabilitation research due to recruitment challenges in specialized populations like active women aged 25–35 years. This may have reduced statistical power to detect smaller effect sizes or subgroup differences, potentially leading to type II errors, and limited generalizability beyond the homogeneous Tehran-based cohort. Small samples are prevalent in physiotherapy trials, often yielding inconclusive results, as noted in meta-analyses of CAI interventions where underpowered studies contributed to heterogeneity. Larger, multicenter trials would enhance external validity [55]. Third, blinding was partial: outcome assessors were blinded, but participants and intervention providers were aware of group allocation due to the tactile nature of FB application, introducing potential performance and detection biases. Participants in the FB group may have experienced heightened expectations or placebo responses, while self-reported measures could be influenced by awareness. This mirrors common blinding issues in CAI studies, where 75% of trials show a high risk of performance bias from unblinded personnel, as per Cochrane reviews. Sham FB protocols (e.g., non-compressive bands) could mitigate this in future designs. Fourth, the lack of long-term follow-up beyond the immediate post-intervention assessment precludes evaluation of sustained effects or recurrence rates. Improvements in balance, JPS, and performance may diminish over time without ongoing adherence, a critical concern in CAI, where 40%–50% of gains from balance training fade by 6–12 months. Studies report that only 42.90% of CAI patients maintain remission at 6 months, underscoring the need for extended tracking to assess durability, as emphasized in recent meta-analyses [56]. These limitations collectively temper the strength of conclusions, advocating for refined methodologies in subsequent research to bolster evidence for FB-augmented PB training in CAI. Mechanistic interpretations (e.g., mechanoreceptor stimulation, reactive hyperemia, BFR-like effects) remain hypothetical; no direct mediator variables were measured. Proximal deficits support inclusion of hop testing [35]. Sex differences in neuromuscular control warrant female-focused protocols [3].

## 5. Conclusions

PB training significantly improved outcomes in women with CAI. FB augmentation was associated with greater short-term gains. These functional improvements support the potential clinical utility of this low-cost adjunct in rehabilitation programs. Larger, sham-controlled, longitudinal studies with mediator assessment are required before inferring injury prevention or long-term joint health benefits.

## Use of AI tools declaration

The authors declare they have not used Artificial Intelligence (AI) tools in the creation of this article.
